# Co-expression network modeling identifies key long non-coding RNA and mRNA modules in altering molecular phenotype to develop stress-induced depression in rats

**DOI:** 10.1038/s41398-019-0448-z

**Published:** 2019-04-03

**Authors:** Qingzhong Wang, Bhaskar Roy, Yogesh Dwivedi

**Affiliations:** Department of Psychiatry and Behavioral Neurobiology, University of Alabama at Birmingham, Birmingham, Alabama 35294 USA

## Abstract

Long non-coding RNAs (lncRNAs) have recently emerged as one of the critical epigenetic controllers, which participate in several biological functions by regulating gene transcription, mRNA splicing, protein interaction, etc. In a previous study, we reported that lncRNAs may play a role in developing depression pathophysiology. In the present study, we have examined how lncRNAs are co-expressed with gene transcripts and whether specific lncRNA/mRNA modules are associated with stress vulnerability or resiliency to develop depression. Differential regulation of lncRNAs and coding RNAs were determined in hippocampi of three group of rats comprising learned helplessness (LH, depression vulnerable), non-learned helplessness (NLH, depression resilient), and tested controls (TC) using a single-microarray-based platform. Weighted gene co-expression network analysis (WGCNA) was conducted to correlate the expression status of protein-coding transcripts with lncRNAs. The associated co-expression modules, hub genes, and biological functions were analyzed. We found signature co-expression networks as well as modules that underlie normal as well as aberrant response to stress. We also identified specific hub and driver genes associated with vulnerability and resilience to develop depression. Altogether, our study provides evidence that lncRNA associated complex trait-specific networks may play a crucial role in developing depression.

## Introduction

The onset of depression is primarily associated with stressful life events, which act as precipitating factors in individuals with increased risk of depression vulnerability [1]. Factors differentiating the phenotypic criteria between susceptibility and resiliency have underscored the involvement of numerous molecular determinants [2]; a majority of them stem out from the complex interplay between the environment and the individual’s genetic makeup [3–5]. In this regard, studies have shown the role of epigenetic variability in shaping the individual’s likelihood of either developing depression or adaptability against depression [6,7,]. Recent studies have demonstrated the influence of system level regulatory network of coding genes and non-coding RNAs in orchestrating a disease specific signature [8,9,]. In this connection, long non-coding RNAs (lncRNA) have shown their critical involvement in disease pathogenesis [10]. LncRNAs are highly expressed in the brain and participate in various normal brain functions as well as disorders, including neurodegenerative and psychiatric illnesses such as schizophrenia, autism, and major depressive disorder (MDD) [11–15]. In addition, a recent peripheral blood profiling study also identified alterations in the expression of lncRNAs in depressed patients [16]. In fact, we and other investigators have shown modifications in small non-coding RNA networks and coding genes in the brain of depressed individuals and animals showing depression-like behavior responsible for coordinating emotional cues and behavioral outcomes [15, 17–20].

Since long-term exposure to stress develops a feeling of helplessness, pervasive sadness, and despair in individuals with a high degree of susceptibility [21], an animal model of stress-induced depression has been developed based on proactive interference with the acquisition of escape or avoidance response when exposed to uncontrollable and unpredictable stress [22]. This model is termed learned helplessness (LH), in which rodents show depression-like behavior, including emotional, cognitive, and motivational deficits [23,24,]. By contrast, the non-learned helpless animals (non-LH/NLH), which are exposed to the same stress, do not show any such behavior (resilient to depression). Thus, this rodent model provides an essential tool to detect the molecular candidates responsible for resiliency vs. vulnerability to developing depression [24]. In this model, we recently showed an overrepresented class of lncRNAs in hippocampus that was uniquely associated with NLH behavior [25]. On the other hand, overall blunted response in lncRNA expression was found to be indicative of possible molecular dysfunctionality associated with underlying gene regulatory network in LH rats [25]. Several other studies have also shown the strong association of specific gene transcripts with LH or NLH behavior [26,27,].

To further examine the role of lncRNAs in stress resiliency and in the vulnerability to develop depression, in the present study we constructed a co-expression network based on differentially expressed mRNAs and lncRNAs in the hippocampus of LH and NLH rats. We also used another group of rats that were not given stress but tested for depressive behavior (tested control, TC) to examine the non-specific effect of behavioral testing per se. To construct such networks, weighted gene correlation network analysis (WGCNA) algorithm [28] was used in which highly correlated lncRNAs or genes based on their expression were clustered into modules [29,30,]. These modules were further analyzed to create a correlation with external traits. In addition, to provide a centrality between connected networks, we identified several probable biological key drivers in the form of “hub” genes [31] specifically associated with the behavior of resilience and vulnerability. Moreover, to explore the relationship between depression-related hub lncRNAs and hub mRNAs, we performed the canonical correlation analysis (CCA), which can detect the linear components that are directly involved in the development of depressive behavior. Thus, our present approach uncovers the underlying molecular mechanisms associated with the susceptibility or resiliency to develop depression and pinpoints potential future therapeutic targets.

## Material and methods

Detailed methods are provided in the Supplementary methods section.

### Animals

All animal experiments were performed in male Sprague–Dawley rats (Holtzman strain) between the age of 6 and 8 weeks under the guidelines of Institutional Animal Care and Use Committee (IACUC) of the University of Alabama at Birmingham. The experimenter was blinded to all the behavioral measurements.

### Induction of learned helpless behavior

**Fig. 1:**
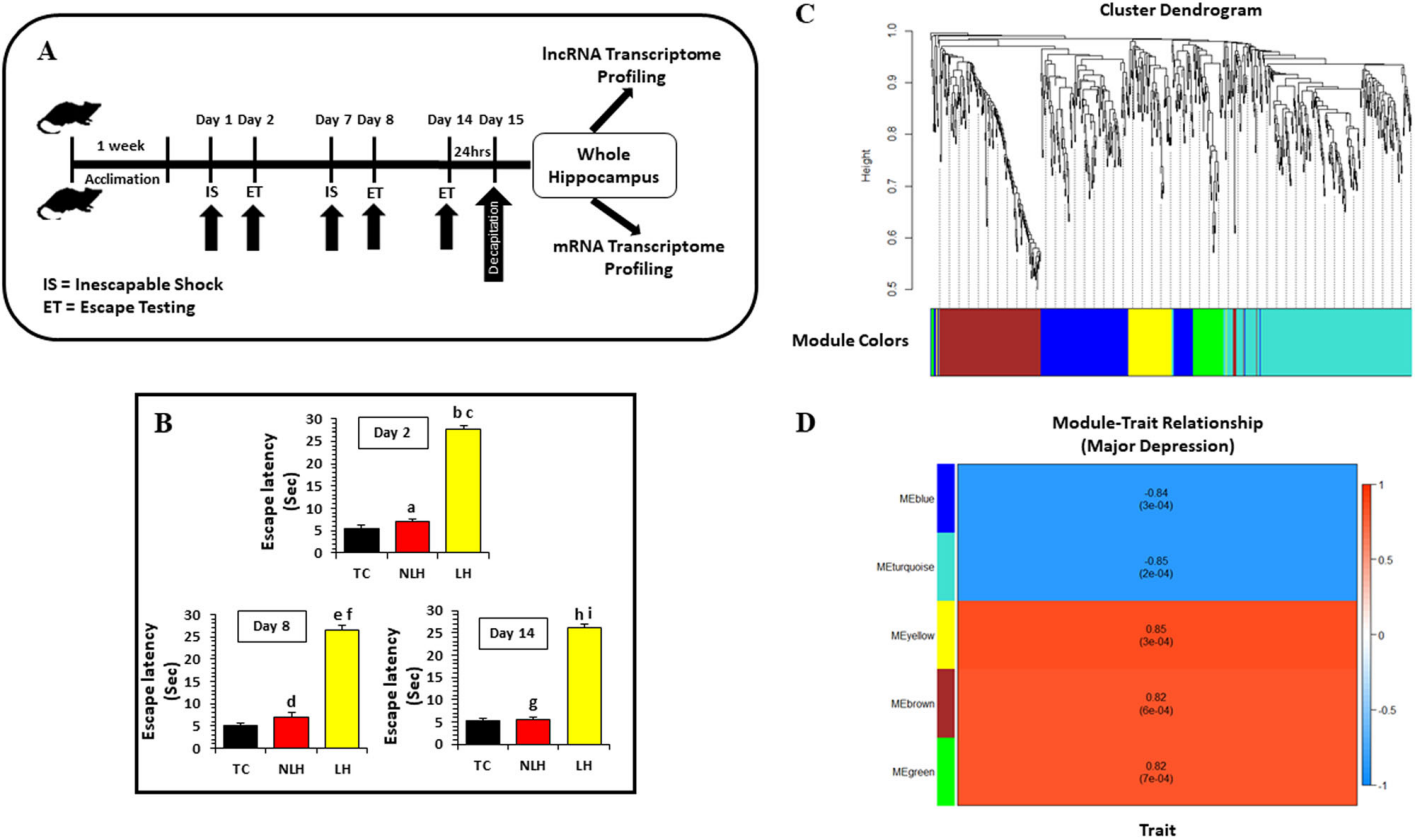
Behavioral paradigm to induce learned helplessness in rat and measuring the effect of shock on their avoidance learning followed by adhering changes in coding genes (mRNA) and their expression-based module assignments to establish a module trait relationship in LH vs. TC rats. **a** Schematic diagram of the timeline followed as part of the stress paradigm to induce LH behavior in rats. **b** Bar diagrams represent escape latencies in tested controls (TC), non-learned helpless (NLH), and learned helpless (LH) rats measured on days 2, 8, and 14, respectively. Data are the mean ± SEM. On day 2, the NLH rats did not show any significant (^a^*p* = 0.15) difference in escape latency compared with the TC group. A significantly (^b^*p* < 0.001) higher escape latency was observed for LH rats compared with TC on day 2. Similarly, LH rats showed significant difference (^c^*p* < 0.001) in mean escape latency compared with the NLH group on day 2. On day 8, NLH rats did not show any significant (^d^*p* = 0.74) difference in escape latency compared with the TC group. A significantly (^e^*P* < 0.001) higher escape latency was noted for LH rats compared with TC rats. Similarly, LH rats showed significant difference (^f^*p* < 0.001) in mean escape latency compared with the NLH group. On day 14, NLH rats did not show any significant (^g^*p* *=* *0.19*) difference in escape latency when compared with the TC group. Individual group comparison identified a significantly (^h^*p* < 0.001) higher escape latency for LH rats compared with TC rats. Similarly, LH rats showed significant difference (^i^*p* < 0.001) in mean escape latency compared with the NLH group. **c** The figure demonstrates the protein-coding gene-based cluster dendrogram analysis in LH vs. TC group. Five colors, which represent the modules, include blue, brown, green, turquoise, and yellow. Dynamic tree cut method was implemented for analysis. The degree of co-expression between the genes assigned by the same module was relatively higher. **d** The figure represents the correlation between mRNAs module eigengenes and phenotypic traits. Each row represents the module eigengene or ME (ME is the correlation matrix of module and sample, labeled by color) and each column represents a trait. Each square contains the Pearson’s correlation coefficient between the MEs and trait and their associated *p* values. The red and blue colors show a strong positive and negative correlation, respectively

The study was performed in learned helpless model of depression in which rats were divided into two groups based on escape latency (ET): LH (vulnerable to depression, showing ET ≥ 20 s; *n* = 7) and NLH (resilient to depression, showing ET < 20 s; *n* = 7). Another group of rats (TC, *n* = 6), which was tested for ET without giving any shock, was also included to rule out the non-specific effects of stress caused by restraint, tail shock, or testing. We used 6–7 rats per group based on our previous study in which we found a robust change in lncRNA expression in hippocampus of LH and NLH rats [25]. The induction of learned helpless behavior is depicted in Fig. 1a, and has been described in the Supplementary methods section (Supplementary information). Twenty-four hours after the final ET test, rats were decapitated, and brains were dissected. Hippocampi were isolated and flash frozen in liquid nitrogen. Tissues were stored at −80 °C until they were analyzed.

#### Microarray-based expression profiling of lncRNAs and mRNAs

The methods for RNA isolation, microarray-expression assay, data capture, and analysis are detailed in the Supplementary method section (Supplementary Information). A schematic diagram (Supplementary Fig. 10) has also been incorporated to represent the overall workflow (including WGCNA-based bioinformatic analysis) associated with this study. Transcriptome-wide lncRNA and mRNA expression was measured using one color high throughput microarray-based microarray protocol using Agilent microarray chip (4 × 44 K) which contained 13,611 lncRNAs and 24,626 mRNAs.

#### Weighted gene co-expression network analysis to determine correlation between lncRNAs and mRNAs

WGCNA was conducted using R software package with WGCNA 1.63 source code. The compilation was done locally after downloading the source code from Comprehensive R Archive Network (CRAN). One additional package, used in the analysis, was downloaded from Bioconductor open source platform [28]. Normalized expression data were used based on lncRNA and mRNA probes. Network construction and module detection were analyzed with the “BlockwiseModules” function in the WGCNA package. Briefly, the Pearson correlation matrix was calculated for all possible RNA pairs and then transformed into an adjacency matrix with soft thresholding power using the “picksoftThreshold” function. A dynamic tree cut algorithm was used to detect groups of highly correlated genes. The minimum module size was set according to the differentially expressed gene (DEG) from each group and the threshold for merging module was set to 0.25 as default. Each module, which was assigned a unique color, contained a unique set of genes.

After obtaining modules from each group, module eigengene, summarized as the first principal component of expression dataset, was calculated with the “ModuleEigengenes” function. The module eigengene is a weighted average of module gene expression profile. Association analysis between a module and the trait of each group was performed as the function of “corPvalueStudent” based on the module eigengene. *p* < 0.05 was set for statistical significance.

The two important parameters, including gene significance (GS) and intramodular connectivity (Ki) were used for identifying hub genes of behavior-associated modules. Intramodular hub genes were selected based on a strong correlation with depression (GSi > 0.9) and higher connectivity (Ki > 0.9). GS_i_ represents the strength of a correlation between a gene and a phenotypic trait. Ki, which means intramodular connectivity, was calculated from the sum of its connection strengths with all the other genes in the same module.

#### Correlation analysis between lncRNAs and mRNAs

To conduct gene-ontology (GO) function for detected modules of lncRNA datasets, we measured the correlation between lncRNAs and mRNAs by Pearson product-moment correlation. We selected the top tenfold change of each module in the lncRNA dataset for correlation analysis. The absolute correlation values were calculated between each lncRNA and the entire DE mRNA dataset in the same group. We ranked absolute correlation values and selected the top ten mRNAs with higher correlation values. For each module of lncRNA dataset, we identified 100 mRNAs highly correlated with lncRNAs for further functional analysis.

#### GO enrichment analysis

GO analysis was explored as the function of each module. We used the “topGo” function in R platform to identify the significant enrichments. The GO category was separated into three groups: the molecular functions, the biological processes (BP), and cellular components. The two-sided Fisher’s test was used to classify the GO category and *p* values were calculated for GOs enriched among different modules. Pathway analysis was performed with the function of “Pathview” and “org.Rn.eg.db” (the rat Genome-wide annotation). The *p* value of the enriched pathway was derived from Database for Annotation, Visualization and Integrated Discovery (DAVID) [32].

We also constructed gene co-expression networks to identify the interactions among genes or lncRNAs of each module. To make a visual network, only the strongest correlations (≥0.94) were included in these representations. The node in the networks represented a coding transcript or lncRNA in the modules. In addition, the two genes connected by an edge indicated a strong correlation (i.e., either positive or negative). The co-expression networks were produced using “igraph” package on the R platform.

#### Specific genes associated with vulnerability (LH) and resilience (NLH)

To specifically identify differentially expressed lncRNAs or mRNAs (DEGs) associated with either vulnerability or resiliency to depression, a Venn diagram analysis was performed with the package “draw.triple.venn” on the R platform. For vulnerability (LH), we subtracted the DEGs overlapping for “LH vs. TC and LH vs. NLH” and overlapping for “NLH vs. TC and LH vs. NLH” from the group of LH vs. TC. For resilience behaviors (NLH), we subtracted the DEGs overlapping for “NLH vs. TC and LH vs. NLH” and overlapping for “LH vs. TC and LH vs. NLH” from the group of NLH vs. TC.

#### Canonical correlation analysis

To explore the relationship between the depression-related hub lncRNAs and hub mRNAs, we performed CCA analysis using the function “CCP” with R from open source code platform GitHub. The underlying principle of CCA is related to multivariate integrative analysis of paired data. Here the CCA can detect the linear components that are directly involved in the development of depressive behavior.

#### First strand cDNA synthesis and qPCR-based transcript quantification of mRNAs

Relative quantification of coding transcripts was determined following the ∆∆Ct method [33] by using first strand complementary DNA (cDNA) synthesized from total RNA. While preparing 1^st^ strand cDNA for coding transcripts, conventional oligo dT priming method was used. Primer sequences are provided in the Supplementary Table 10. For all quantitative polymerase chain reaction (qPCR)-based expression studies, four to seven animals were used as biological replicates.

## Results

### Behavioral analysis by testing escape latencies

One-way ANOVA, followed by post hoc test, determined significant difference in escape latency (EL) (*p* < 0.01) between TC, NLH, and LH groups when tested on three different days (day 2: d*f* = 2,17, *F* = 182.04; day 8: d*f* = 2,17, *F* = 381.86; day 14: d*f* = 2,17, *F* = 277.23). Individual group comparison showed significantly higher EL for LH rats compared with TC and NLH rats on days 2 (*p* < 0.001), 8 (*p* < 0.001), and 14 (*p* < 0.001). NLH rats did not show any significant difference in EL when compared with the TC group at any time point (day 2: *p* = 0.15; day 8: *p* = 0.74; day 14: *p* = 0.19) (Fig. 1b).

### WGCNA analysis using mRNA-expression data

We first conducted module detection analysis in the mRNA datasets across the three groups (TC, LH, and NLH). We implemented the dynamic branch-cutting algorithm with a robust measure of interconnectedness using DynamicTreeCut and the WGCNA R library. Each module was assigned a unique color label, which is visualized underneath the cluster dendrogram shown in Fig. 1c. The most striking mRNA response was observed in the LH vs. TC comparison. The total number of 756 DEGs reached the criteria where *p* value was <0.05 and the fold change was >1.3 (Table 1). WGCNA among LH vs. TC revealed five modules: LTGblue, LTGturquoise, LTGyellow, LTGbrown, and LTGgreen. Module association analysis demonstrated that LTGblue (*r* = −0.84, *p* = 3E−04, *n* = 175) and LTGturquoise (*r* = −0.85, *p* = 2E−04, *n* = 290) modules were negatively correlated with phenotype changes within the two groups. This shows that these two modules comprised of genes that have higher expression levels in the LH group. In contrast, the other three modules: LTGyellow (*r* = 0.85, *p* = 3E−04, *n* = 71), LTGbrown (*r* = 0.82, *p* = 6E−04, *n* = 168), and LTGgreen (*r* = 0.82, *p* = 7E−04, *n* = 52) showed positive correlation (Fig. 1d).

**Table 1:** Module–trait relationship associated with differentially expressed lncRNAs and mRNAs in learned helpless (LH), non-learned helpless (NLH), and tested control (TC) groups

Type	DEGs	Module	Genes	Analysis summary					
				GO	Pathway	Network	Hub gene	*R* value	*p* Value
LNG (LH vs. NLH mRNA microarray)		Blue	158	√	√	√	3	−0.79	9.00E−04
	417	Brown	16	√	√	√	1	−0.7	5.00E−03
	Up: 355; down:62	Turquoise	242	√	√			−0.76	1.00E−03
LTG (LH vs. TC mRNA microarray)		Blue	175	√	√			−0.84	3.00E−04
		Brown	168	√	√			0.82	6.00E−04
	756	Turquoise	290	√	√	√	3	−0.85	2.00E−04
	Up: 458; down: 298	Green	52	√	√	√	2	0.82	7.00E−04
		Yellow	71	√	√			0.85	3.00E−04
NTG (NLH vs. TC mRNA microarray)		Blue	55	√	√			−0.8	9.00E−04
		Brown	17	√	√			0.79	1.00E−03
	395	Turquoise	316	√	√	√	1	0.83	4.00E−04
	Up: 135; down: 260	Yellow	7	√	√			−0.66	1.00E−02
LNC (LH vs. NLH lncRNA microarray)		Blue	18	√	√			−0.71	4.00E−03
	314	Brown	6	√	√			0.7	5.00E−03
	Up: 62; down: 252	Turquoise	315	√	√	√	4	−0.82	3.00E−04
LTC (LH vs. TC lncRNA microarray)		Blue	226	√	√			−0.89	2.09E−05
	729	Brown	166	√	√	√	7	0.87	2.45E−03
	Up: 346; down: 383	Turquoise	270	√	√	√	13	0.87	2.76E−04
		Yellow	67	√	√			0.8	8.41E−04
NTC (NLH vs. TC lncRNA microarray)		Blue	32	√	√			−0.76	3.00E−03
		Brown	22	√	√			−0.77	2.00E−03
	443	Turquoise	364	√	√	√	6	0.8	1.00E−03
	Up: 359; down: 84	Yellow	11	√	√			0.69	9.00E−03
		Red	6	√	√			−0.76	3.00E−03
		Yellow	8	√	√			0.69	9.00E−03

A total of 395 DEGs were significantly altered in the NLH vs. TC comparison. Four modules were found in this group comparison: NTGbrown, NTGturquoise, NTGblue, and NTGyellow (Supplementary Fig. 1A). Because we focused only on the differentially expressed mRNAs, the four modules showed significance with phenotypic differences and the blue module was most significantly associated with resilience (NLH). The brown and turquoise modules were positively correlated while the blue and yellow modules were negatively correlated (Supplementary Fig. 1B).

In the LH vs. NLH comparison, 417 DEGs were observed which included 355 upregulated and 62 downregulated mRNAs. Three modules were detected: LNGturquoise, LNGblue, and LNGbrown. Interestingly, all three modules showed negative correlation with phenotypic outcome (Supplementary Fig. 2A, B).

### Functional annotations of mRNA co-expression networks

**Fig. 2:**
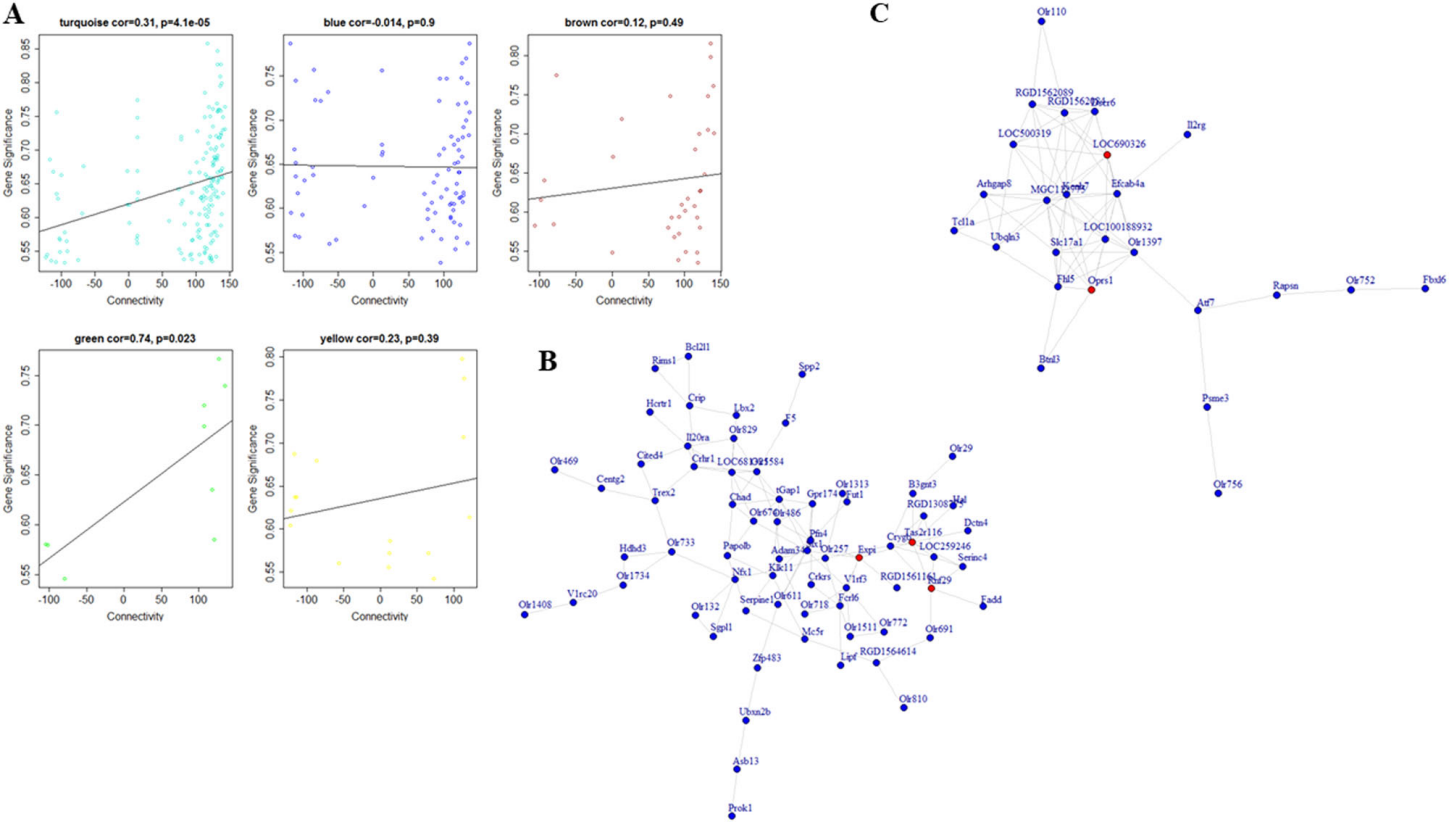
Identification of intramodular connectivity and gene significance supported by network analysis. **a** The correlation analysis between intramodular connectivity and gene significance for five independent modules are individually represented with scatterplot. The intramodular connectivity of two modules containing turquoise and green was significantly correlated with gene significance. **b** The igraph generated network-based visualization demonstrates the hub genes from the “Turquoise module” associated with susceptibility phonotype. The red dots represent the hub genes which include *Expi*, *Tas2r116*, and *Rnf29*. **c** The figure represents the hub genes associated with “Green module”-based network. The two hub genes from this network, *LOC690326* and *Oprs1*, are also represented with red dot

We used topGO, Pathview, and DAVID to investigate gene ontology and pathways across each module. For the LH vs. TC group, LTGturquoise, LTGblue, and LTGbrown were the top three modules (considering the number of genes enriched for each module) that were significantly associated with LH behavior. The relatedness of traits with significantly altered hub genes often comprise an intramodular connectivity within the network module and represent a convenient course to pathway-based gene screen procedure depending on the degree of intramodular connectivity. As shown in Fig. 2a, the LTGturquoise (*p* = 4.1E−05) and LTGgreen (*p* = 0.023) modules showed significant correlations between intramodular connectivity and gene significance. The LTGturquoise module was by far the largest module containing 290 transcripts. GO-enrichment analysis showed that among others, the biological process related to “G-protein coupled receptor (GPCR) signaling pathway” and “detection of chemical stimulus” involved in sensory perception were most significantly associated with turquoise module (Supplementary Table 1). The pathway analysis indicated that the two pathways most significantly enriched among various KEGG pathways were (1) olfactory transduction (*p* = 4.30E−08) associated with 44 DEGs and (2) neuroactive ligand–receptor interaction (*p* = 3.10E−02) (Supplementary Table 2). The hub mRNAs in the LTGturquoise module were: *Expi*, *Rnf29*, and *Tas2r116* (Fig. 2b), which were upregulated in the LH group (*Expi*: fold change = 1.645, *p* = 3.78E−03; *Rnf29*: fold change = 1.830. *p* = 1.93E−03; *Tas2r116*: fold change = 1.984, *p* = 9.22E−04) (Supplementary Table 3). The network diagram of the LTGturquoise module demonstrates that *Expi*, *Rnf29*, and *Tas2r116* have strong intramodular connectivity with other genes (Fig. 2b).

The LTGgreen module consisted of 52 DEGs (Table 1). The network diagram of LTGgreen is shown in the Fig. 2c. The GO and pathway analysis indicated that the function of LTGgreen module was very close to that of turquoise modules. Both were associated with olfactory transduction (Supplementary Tables 1 and 2). Two hub genes of LTGgreen module were *Sigmar1* and *Lpal2*. Both of them were significantly downregulated (*Sigmar1*: fold change = 1.454, *p* = 2.07E−03; *Lpal2*: fold change = 1.561, *p* = 3.62E−02) in the LH compared with the TC group (Supplementary Table 3). Both of them are known for their roles in CNS disorders including Alzheimer’s disease [34].

The LTGblue module consisted of 175 DEGs. Unlike the LTGturquoise and green modules, this module did not show significant correlation (*p* = 0.9) between intramodular connectivity and gene significance (Fig. 2a). The GO and pathway analysis indicated that the function of LTGblue module was close to that of the turquoise module. The GO analysis for LTGblue module showed enrichment for the BP related to “GPCR signaling pathway” and “response to chemical stimuli” (Supplementary Table 1). Like LTGturquoise, LTGblue module was also associated with olfactory transduction (*p* = 9.10E−03) and neuroactive ligand–receptor interaction (*p* = 3.70E−03) pathways (Supplementary Tables 2). Next, in the LH vs. TC group comparison, the LTGbrown module was not able to show any significant correlation (*p* = 0.49) between intramodular connectivity and gene significance (Fig. 2a). The biological process of LTGbrown module (DEGs: 168) in the GO analysis was related to the “negative regulation of APC-Cdc20 complex activity,” “multi-organism reproductive process,” and “regulation of cell cycle” (Supplementary Table 1). Pathway analysis found that olfactory transduction (*p* = 8.50E−03) and viral myocarditis (*p* = 6.10E−02) pathways were the most enriched ones (Supplementary Table 2). Like LTGblue and brown modules, the yellow module did not have any significant correlation between intramodular connectivity and gene significance (*p* = 0.39) (Fig. 2a). Moreover, none of the associated pathways (total seven pathways considered) for this module were statistically significant (Supplementary Table 2).

The two modules, NTGblue and NTGturquoise showed the most significant association with the resilience (NLH) (Supplementary Fig. 1B). Nevertheless, only NTGturquoise connectivity was significantly associated with gene significance (*p* = 0.0011) (Supplementary Fig. 3A). NTGturquoise network is shown in Supplementary Fig. 3B. As shown in Supplementary Fig. 3B and Supplementary Table 3, *My13* gene encoding protein: myosin light chain 3 was the hub mRNA in the NTGturquoise module with a downregulated expression (fold change = 2.079, *p* = 6.58E−03), whereas the pathway associated with “olfactory transduction” was represented by both NTGturquoise and NTGblue modules (Supplementary Table 2).

Both LNGblue and LNGbrown modules from LH vs. NLH comparison group showed significant correlation between intramodular connectivity with gene significance (LNGblue: *p* = 0.0028; LNGbrown: *p* = 0.031) except LNGturquoise (*p* = 0.091) (Supplementary Fig. 4A). The LNGblue module, included three hub genes, i.e., *Inexa* (internexin neuronal intermediate filament protein), *Olr8* (olfactory receptor 8), and *Sgpl1* (sphingosine-1-phosphate lyase 1) (Supplementary Fig. 4B and Supplementary Table 3) with potential neurobiological functions. These three mRNA transcripts were significantly upregulated in the LH relative to NLH group. The LNGblue module also showed enriched olfactory transduction. For the LNGturquoise module, *Fadd* (coded with Fas associated via death domain) was identified as hub mRNA, which seems to play a crucial role in the entire network (Supplementary Figure 4C). The olfactory transduction was also enriched in the LNGbrown module. In summary, the genes in the olfactory transduction pathway appear to be directly involved in the phenotypic differences between LH and NLH groups (Supplementary Table 2).

### WGCNA analysis using lncRNA-expression data

**Fig. 3:**
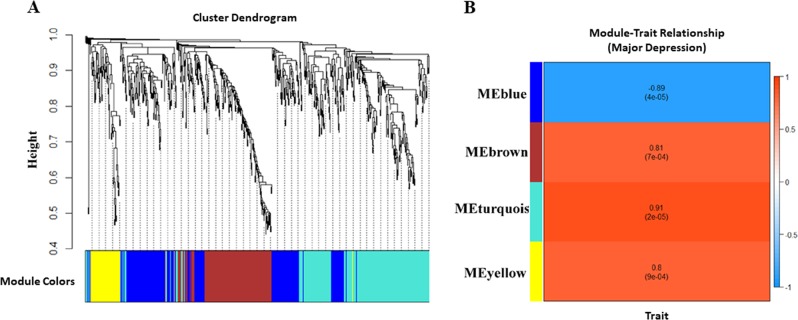
LncRNA expression-based module assignments and module trait relationship in LH vs. TC groups. **a** Using topological overlapping matrix dissimilarity, the cluster dendrogram was prepared to show four individual modules including LTCblue, LTCbrown, LTCturquoise, and LTCyellow. **b** The representative figure demonstrates the correlation analysis between the four modules and depression phenotype. Unlike the three other positively correlated modules (LTCbrown, LTCturquoise, and LTCyellow), the LTCblue module shows negative correlation with depression (LH) phenotype

As with mRNA studies, lncRNA datasets were used to compare LH vs. TC, NLH vs. TC, and LH vs. NLH groups. Among the 3 comparisons, the most notable changes were in the LH group relative to the TC group, as we found 729 differentially regulated lncRNAs, including 346 up and 383 downregulated (Table 1). With these 729 lncRNAs, WGCNA provided four modules: LTCblue LTCbrown, LTCturquoise, and LTCyellow. However, only LTCblue module showed negative association with the LH phenotype (Fig. 3a, b). LH vs. NLH comparison showed 314 DEGs. Among them, 62 were upregulated and 252 were downregulated (Table 1). A cluster dendrogram of the co-expression modules is shown in Supplementary Fig. 5A. Three modules were observed based on the topology network analysis: LNCbrown, LNCblue, and LNCturquoise, which demonstrated a statistically significant association with NLH phenotype (Supplementary Fig. 5B). The brown module was positively associated with changes in phenotypic trait, whereas blue and turquoise modules were negatively correlated. In Supplementary Fig. 6A, 6B, the dendrogram plot, associated modules, and differentially regulated lncRNAs in each module are demonstrated for NLH vs. TC group. Five individual modules (NTCgreen, NTCturquoise, NTCyellow, NTCred, NTCblue, and NTCbrown) were identified based on lncRNA expression profile. NSCturquoise was identified as the most significantly associated module with resiliency (NLH).

### Functional annotations of lncRNA co-expression networks

To annotate the function of different lncRNA modules, we examined the co-expression correlations between lncRNA and mRNA in the same group. We selected top ten lncRNAs with highest fold change as representative of the module. We further conducted the correlation analysis between lncRNAs and coding genes (mRNA). Top ten mRNAs having the highest correlations with lncRNAs were selected for functional annotations. Some lncRNAs had the overlapping correlations with the same mRNAs. Thus, the number of mRNAs was less than 100 within each module.

**Fig. 4:**
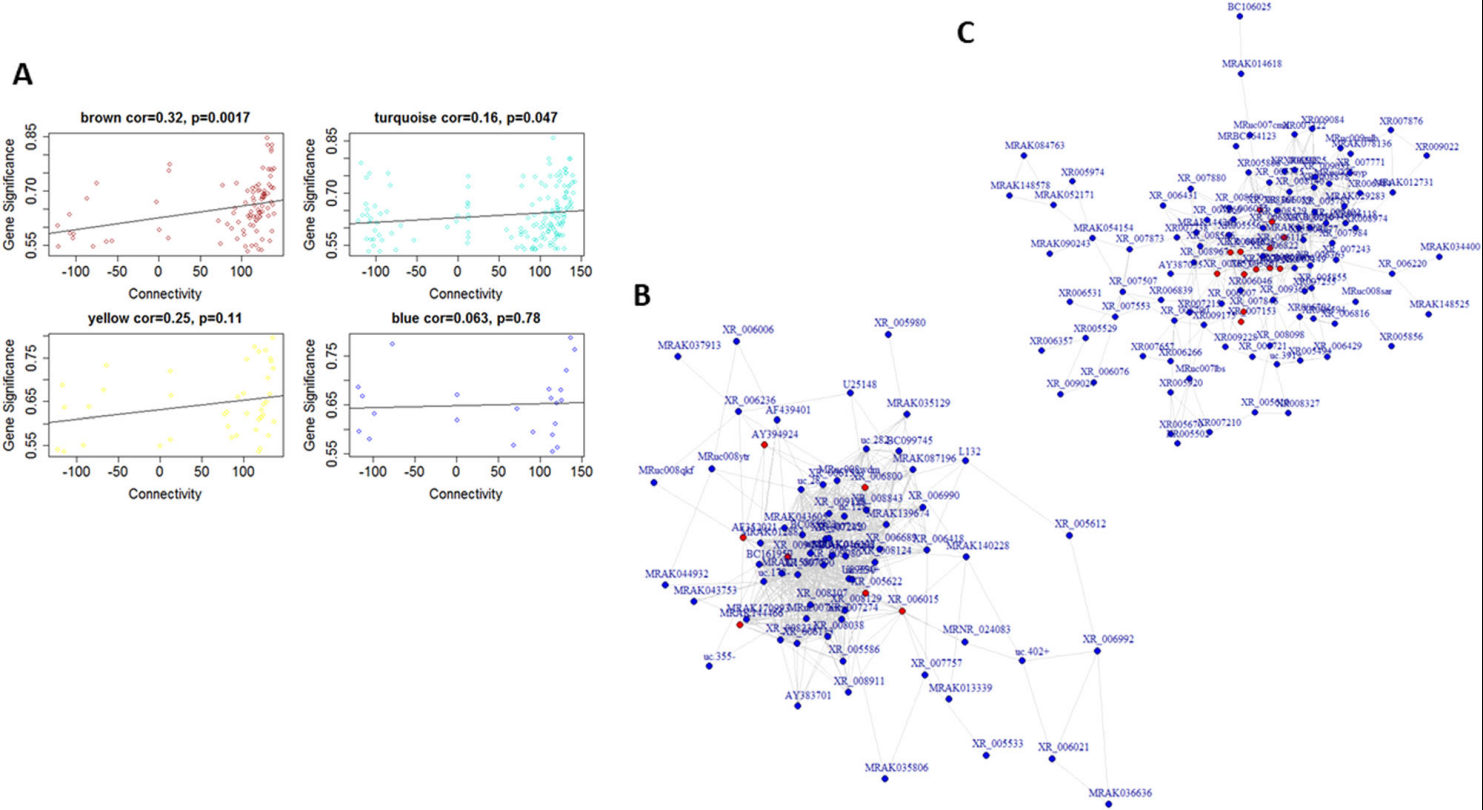
Correlation analysis for gene significance and scaled connectivity mapped with hub lncRNAs in the network. **a** The scatterplots represent the relationship between gene significance and connectivity based on lncRNA associated expression changes in LH vs. TC groups. The correlation between gene significance and connectivity are associated with LTCbrown (*p* = 0.0017) and LTCturquoise (*p* = 0.047) modules. **b** The figure represents network associated with LTCbrown module with connectivity to lncRNA transcribing hub genes. **c** The figure represents the hub genes associated with “Turquoise module”-based network

For the LH vs. TC group comparison, LTCblue module had significant enrichment of genes related to “GPCR signaling,” “signal transduction,” and “sensory perception of chemical stimulus.” On the other hand, the LTCbrown module was overrepresented by genes associated with “dopamine transport,” “monoamine transport,” and “maternal behavior.” Another important module, LTCtuquoise, seems to play an important role in the “actin–myosin filament sliding,” “trans-Golgi network transport vesicle,” “core promoter binding,” and “RNA polymerase II transcription factor associated functions.” The detailed information is listed in Supplementary Table 4. Pathway analysis demonstrated that riboflavin metabolism in the LTCblue (*p* = 7.50E−02) and cardiac muscle contraction in the LTCbrown (*p* = 3.70E−02) and LTCturquoise (*p* = 1.80E−02) were associated with LH (Supplementary Table 5) phenotype. We also identified 20 hub lncRNAs in the LH vs. TC comparison (Supplementary Table 6). The network analysis illustrated significantly higher interconnectivity of two modules: LTCturquoise (*r* = 0.16) and LTCbrown (*r* = 0.32) (Fig. 4a). Therefore, LH behavior appears to be more complex and caused by the cohesive connectivity of modules rather than by a single gene (Fig. 4b, c).

For the NLH vs. TC comparison, NTCturquoise module was identified as having strong correlation between gene significance and connectivity (*p* = 0.0039) (Supplementary Fig. 7A). The GO analysis for NTCturquoise module is shown in Supplementary Table 4. Interestingly, the olfactory transduction pathway was highly enriched in the NTCblue module (*p* = 3.50E−02) of the NLH group (Supplementary Table 5). Six hub lncRNAs from NTCturquoise were found to be significant: XR_008967, XR_008566, XR_009175, XR_007909, XR_006115, and XR_005783 (Supplementary Table 6, Supplementary Fig. 7B).

For the LH vs. NLH group, LNCturquoise module showed significant correlation between gene significance and connectivity (*p* = 0.00017) and was comprised of 314 DE lncRNAs (Supplementary Fig. 8A). Functional annotation showed that LH vs. NLH was significantly (*p* = 1.60E−03) associated with olfactory transduction pathway (Supplementary Table 5). We found four hub lncRNA in the LNCturquoise module, including *AY539919*, *AY562215*, *MRAK039538*, and *MRAK048306* (Supplementary Fig. 8B). The information about hub lncRNAs is listed in Supplementary Table 6.

### Specific analysis

**Fig. 5:**
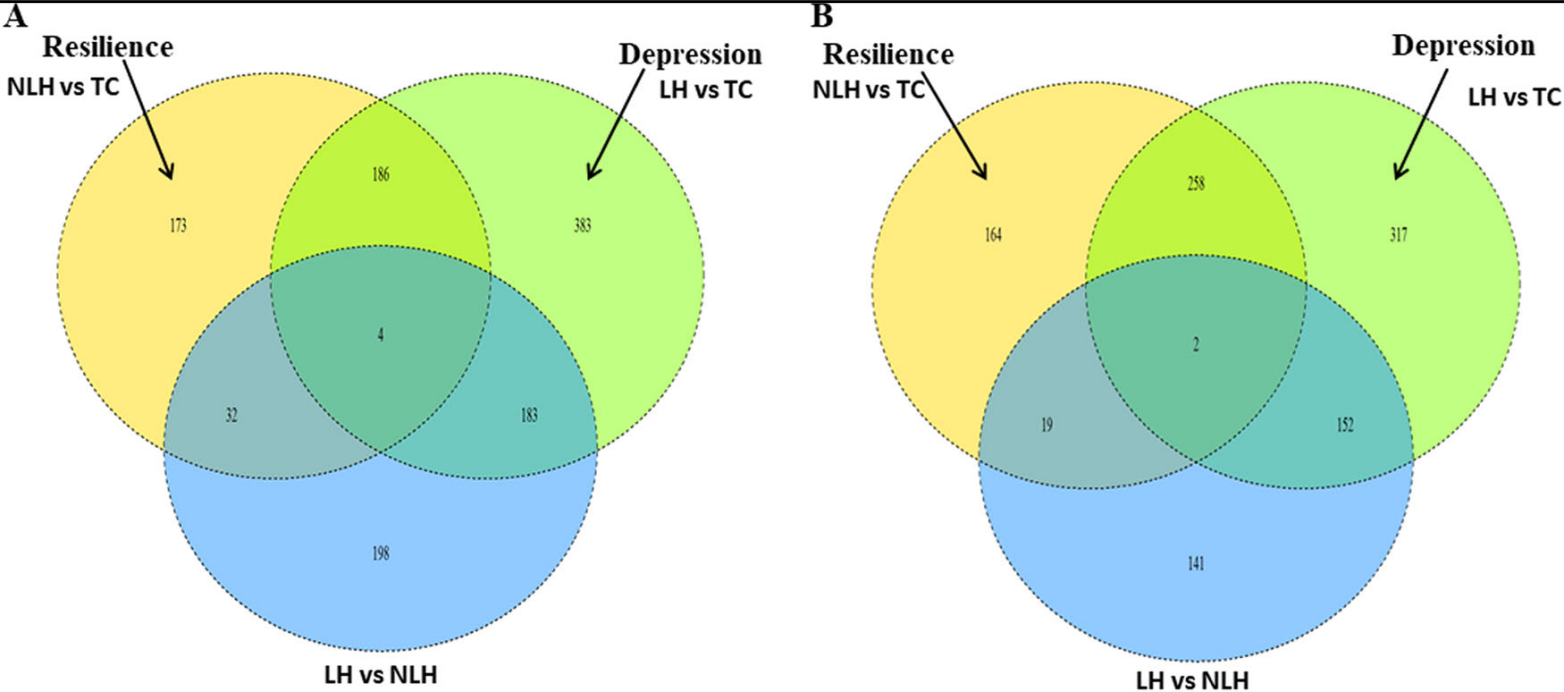
Venn diagram of overlapping mRNA and lncRNA derived from three group comparisons. **a** Differentially expressed mRNAs are represented on this Venn diagram, showing either distinct or overlapping relationship with depression or resiliency phenotype. **b** The diagram represents the unique and overlapping phenotypic association of differentially expressed lncRNA with resiliency or susceptibility to develop depression

To identify the overlapping and phenotype-specific genes, a Venn diagram was generated, which showed that 383 mRNAs (Fig. 5a) and 317 lncRNAs (Fig. 5b) were specifically associated with vulnerability to depression (LH behavior). On the other hand, the 173 mRNAs and 164 lncRNAs were specifically associated with resiliency to depression (NLH group) (Fig. 5a, b). Among the 383 mRNAs specific to LH group, *olr510* (olfactory receptor 510, *p* = 0.0017, fold change = 2.74), *Sptlc3* (serine palmitoyl transferase, *p* = 0.000001, fold change = 2.72), *Sult2a2* (sulfotransferase family 2A, fold change = 2.375; *p* = 0.015), *Six1* (SIX homeobox 1) (fold change = 2.277; *p* = 0.0103), and *Agxt2* (alanine-glyoxylate aminotransferase 2, fold change = 2.256, *p* = 0.0136) were the top five ranked genes (Supplementary Table 7). On the other hand, out of 183 short-listed genes from NLH vs. TC group, *Pnpt1* (*p* = 0.0001, fold change = 1.534), *Lmo2* (*p* = 0.0006, fold change = 1.353), *Tmem163* (*p* = 0.0007, fold change = 1.569), *B3gnt8* (*p* = 0.0007, fold change = 1.759), and *Sfrs14* (*p* = 0.0009, fold change = 1.310) were the top significantly altered coding transcripts (Supplementary Table 8) specific to resilience phenotype (NLH). Furthermore, we found that olfactory transduction was enriched both in the susceptibility (Supplementary Table 7) (LH) and resiliency (NLH)-related genes (Supplementary Table 8).

### Canonical correlation coefficient analysis (CCA)

The canonical correlation coefficients (CCA) are shown in Supplementary Table 9. The CCA was restricted to five factors because the hub mRNA set contained five variables. The canonical correlation between variate-1 hub lncRNAs and mRNAs were statistically significant (*p* = 0.0085) without any change in other variates (variats 2–5).

### qPCR-based analysis of select hub genes in hippocampus of TC, NLH, and LH rats

We selected four hub genes (*Rnf29*, *Tas2r116*, *Myl3*, *Inexa*, and *Olr8*) across various comparison groups and analyzed their expression by qPCR using the RNA isolated from hippocampus. It was found that the two hub genes (*Rnf29* and *Tas2r116*) from TC vs. LH group were significantly upregulated (*Rnf29*, *p* = 0.04; *Tas2r116*, *p* = 0.02) similar to that observed in the microarray analysis (Supplementary Fig. 9A). On the other hand, two hub genes *Inexa* and *Olr8* were upregulated in LH vs. NLH group, as in microarray analysis (Supplementary Fig. 9C); however, they were not statistically significant.

## Discussion

In a previous study, we had examined the expression of lncRNAs in the hippocampus of LH, NLH, and TC rats and found that a large number of lncRNAs were differentially regulated in LH and NLH groups [25]. In the present study, we constructed the weighted gene co-expression networks based on the differentially expressed mRNAs and lncRNAs in three groups of rats with different behavioral phenotypes. First, we statistically analyzed the differentially expressed mRNAs and lncRNAs by considering their fold change and *p* value derived from the microarray-based expression data. Differentially expressed lncRNA and mRNA data from three groups were used to detect expression modules. We further calculated the relationship between specific modules and phenotype of each comparison group. Following a guilt-by-association approach, candidate genes with close association to phenotypic changes were also identified in each comparison and designated as hub genes. Additional analysis was conducted to determine the related GO and pathways based on identified expression modules and hub genes. Due to the limited knowledge about lncRNA functions in rat species, we integrated the lncRNA and mRNA microarray data to detect correlations between them.

In our study, we conducted transcriptome-wide expression array using 8977 lncRNA and 14605 mRNA probes and identified differentially expressed transcripts with fold change > 1.3 and *p* value < 0.05 in LH vs. NLH, LH vs. TC, and NLH vs. TC groups. Among the three groups, the number of differentially expressed lncRNAs (*n* = 729) was more in the LH vs. TC group than in the other two comparing groups. Interestingly, similar to lncRNAs, mRNA dysregulation in LH vs. TC was the most significant among the three groups. The striking alteration of transcripts in LH vs. TC was found to be associated with different phenotypic changes. Among all 458 upregulated mRNA transcripts in LH vs. TC, the “*Olr60*” related to olfactory receptor 60, showed the highest expression change. On the other hand, out of 298 downregulated transcripts, *Olr601* coded by olfactory receptor 601 was found to be strikingly downregulated in this group. Similarly, our analysis in the NLH vs. TC group identified *Txndc8* (thioredoxin domain containing 8), which was the top ranking mRNA based on the magnitude of its expression. In the LH vs. NLH comparison, we identified several interesting mRNAs including S*lc10a5* (solute carrier family 10, member 5), *Vom1r42* (vomeronasal 1 receptor 42), and *Olr1222* (olfactory receptor 1222), primarily for their potential involvement in the central nervous system (CNS)-related functions [35–37]. We also tested qPCR-based expression of a few selected hub genes. As elaborated in the results section, we were able to replicate the findings observed in the microarray experiments. Although we only found significant changes for *Rnf29* and *Tas2r116* driver genes, unlike the other three tested genes (*Myl3*, *Inexa*, and *Olr8*) from TC vs. NLH and LH vs. NLH groups, they showed a similar pattern of expression. This validates our findings from microarray expression assay regarding hub gene identification.

The DEG extracted from the gene-expression array experiment provided interesting results considering their potential value as biomarkers for mental illnesses. In the past, compelling evidence has suggested that individual genes only play a partial role in the development and progression of psychiatric phenotype. Increasing number of studies have suggested that complex phenotypic disorders are associated with a correlation between genes and are often supported by intricate co-expressional network, which connects clinical traits, genome and transcriptome [14,38,]. To construct such networks, WGCNA algorithm [28] is one of the potential tools used frequently in studies to correlate gene expression into clustered modules. WGCNA analysis has been used in many neuropsychiatric studies [39–41]. In a recent study, specific gene modules were obtained from depressed patients and found to be significantly associated with clinical improvement [42]. GO analysis suggests that specific modules were enriched in the inflammatory and immune pathways [42]. More recently, the use of WGCNA has enabled the characterization of sexually dimorphic gene networks conferring sex-specific stress susceptibility in the depressed brain [43]. In our study, we also identified several modules from the lncRNAs and mRNAs in each comparison and found that each identified module was significantly associated with phenotypic changes representing specific behavior. Interestingly, we also found that all the mRNA modules (blue, brown, and turquoise modules) in the LH vs. NLH comparison were negatively associated with resiliency to depression.

Interestingly, pathway analysis demonstrated that several modules from LH vs. TC comparison (blue, brown, turquoise, and green) and LH vs. NLH comparison (blue, brown, turquoise; and blue and turquoise) were significantly associated with olfactory transduction. Also, GO analysis suggested that these modules were significantly associated with sensory abilities. Based on these findings, it can be strongly hypothesized that dysfunctionality in olfactory processing might be associated with the development of depression. In fact, removal of olfactory bulb (OB) induces changes in neurochemical, neuroanatomical, physiological, and endocrine functions [44]. Previous research examining mouse models of chronic stress and human hippocampal structure in MDD has found a reduction in hippocampal volume and an abnormality in hippocampal neurogenesis [45,46,]. These alterations are likely due to changes in glucocorticoid levels following chronic stress [47]. In fact, the use of rodent models have demonstrated that deficits in OB functioning can impact the hippocampus and can induce depressed mood [44]. Research has shown that olfactory bulbectomy leads to anhedonia and behavioral changes, combined with deficits in spatial learning, avoidance learning, conditioned taste aversion and food-motivated behaviors, which frequently resemble symptoms seen in human patients with major depression [48]. Antidepressant treatment reverses the depressive behavior of olfactory bulbectomized rats [49,50,]. Rats subjected to unpredictable chronic mild stress show altered gene expression and signal transduction pathways in the OB [44,48,51,]. Clinical studies have also demonstrated that depressed patients show a marked reduction in the sensitivity to olfactory cues; the extent of the reduction is associated with the severity of the symptoms [52]. From an evolutionary perspective, the connection of OB is well recognized in the limbic system [53]. Therefore, a change in OB can cause abnormality in emotional cues by affecting the prefrontal limbic network of the stressed brain [54]. The cognitive theory of depression proposes that MDD is associated with a negative bias in thinking [55]. The application of this theory may be partly due to neuroanatomical projections between the OB and the limbic system that can impact sensory functions and olfactory processes in depressed individuals [56]. Studies have shown that decrease in potential amplitudes of the chemosensory impulses in MDD patients reflect a modality-specific reduction in the ability to encode basic olfactory information. This modality-specific change is more prominent in MDD than other psychiatric disorders [52]. Based on our current findings, these neuroanatomical and neurochemical changes can be interpreted well at the genetic level. In addition, implying the role of complex genetic regulatory network may help in understanding the epigenetic involvement of lncRNA with this sensory-cognitive dysfunctionality at system level.

Next, we integrated the data from three comparisons and selected the candidate genes specific to depression vulnerability or resiliency. Interestingly, the enrichment of olfactory transduction pathway appeared again both in the susceptibility and resiliency. Our results also revealed a significant correlation between lncRNAs and mRNAs using the CCA, which elucidated their potential regulatory mechanisms. A similar correlation between the lncRNA- and mRNA-expression profiling has recently been reported in the peripheral blood of depressed patients [16].

Hub genes play a central role in the co-expression networks. For the LH vs. TC comparison, 5 hub mRNAs (*Tas2r116*, *Expi*, *Rnf29*, *Oprs1*, and *LOC690326*) and 20 lncRNAs were identified as hub genes. *Tas2r116* is a member of vertebrate taste receptor family and represents the GPCR superfamily [57]. Interestingly, a previous study in rats linked restraint stress with diminished expression of one of the mRNA subunits of the sweet taste receptor (*Tas1r3*) in taste tissue and reduced gustatory nerve excitation by sweet compounds [58]. Others found that receptors for stress-activated hormones are localized in the oral taste cells and glucocorticoids may act directly on taste receptor cells under stressful conditions and determine how these cells respond to taste stimuli [59]. *Expi* is located on chromosome 10q26, includes three exons encoding for extracellular proteinase inhibitors, and is involved in the proteinase inhibiting capacity, cell invasive, and cell metastatic potential [60,61,]. RNF29 (TRIM55), as a member of TRIM family, functions as Ubiquitin E3 ligase enzyme and regulates the degradation of target proteins. RNF29 is associated with the modulation of innate immunity [62], and its homologous family members are known for their role in neuropsychiatric abnormalities, including schizophrenia, attention deficit hyperactivity disorder and X-linked intellectual disability [63]. Besides this, one hub mRNA (*Myl3*) and four lncRNAs in the LH vs. NLH group, four mRNAs (*Inexa*, *Olr8*, *Sgpl*, and *Fadd*) and six lncRNAs in the NLH vs. TC group were analyzed with the stringent criteria. These hub genes were not only highly correlated, but also had high-intramodular connectivity with the other genes in the same module. At present, it is difficult to illustrate the biological functions of these hub genes; however, they indicate new molecular pathways that can be associated with resiliency or development of depression phenotypes. Further studies will be needed to dissect their phenotypic association.

In conclusion, based on the mRNA and lncRNA microarrays, we found signature co-expression networks that underlie normal as well as aberrant response to stress. We constructed modules and analyzed the association between the modules with different phenotypes. These modules were enriched in the olfactory transduction, neuroactive ligand–receptor interaction, etc. We also identified hub and specific driver genes associated with vulnerability and resilience. Altogether, our study provides solid evidence that these complex trait-specific networks may play a crucial role in resiliency or vulnerability to develop depression. However, one has to be cautious in interpreting these findings in humans as rodents are adapted to use more sensory functions which results in much larger OB size (200-fold) compared to humans. Although it is difficult to translate rodent findings to humans, the association of lncRNA in major depression has been elaborated in two recent studies using human subjects. One study, conducted in peripheral blood, showed the differential regulation of both lncRNAs and mRNAs in MDD patients, which were mostly associated with metabolic process and neurodevelopmental disorders [16]. Another study, conducted in the anterior cingulate cortex of depressed subjects who had died by suicide, showed significant changes in the expression of a large number of lncRNAs and their possible regulatory roles in altering transcriptome dynamics implicated in various molecular pathways associated with depression such as cytoskeleton organization, plasma membrane, cell adhesion, nucleus, DNA-binding, and regulation of dendrite development and morphology [15]. In our earlier study, we found the role of lncRNAs in RNA transport, mRNA surveillance, metabolic processes, intercellular communications, and anatomical structure maintenance in the LH rats [25], which are similar to what has been shown in humans. Further studies will be needed to identify the specific involvement of lncRNAs in depression pathogenesis. In addition, it will be interesting to study other brain areas besides hippocampus to examine if similar or different lncRNAs and corresponding target genes are associated with susceptibility to depression.

## Supplementary information

Supplementary Information

Supplementary Table 1

Supplementary Table 2

Supplementary Table 3

Supplementary Table 4

Supplementary Table 5

Supplementary Table 6

Supplementary Table 7

Supplementary Table 8

Supplementary Table 9

Primer sequences used for qPCR expression assay

Supplementary Figure 1

Supplementary Figure 2

Supplementary Figure 3

Supplementary Figure 4

Supplementary Figure 5

Supplementary Figure 6

Supplementary Figure 7

Supplementary Figure 8

qPCR-based expression changes of select hub genes associated with rat model of depression

Illustration of the workflow analysis followed for data generation
