# Supplementary material for: Co-expression network modeling identifies key long non-coding RNA and mRNA modules in altering molecular phenotype to develop stress-induced depression in rats
Source: Transl Psychiatry. 2019 Apr 3;9:125. doi: 10.1038/s41398-019-0448-z (PMC6447569; doi:10.1038/s41398-019-0448-z)
Supplement: Supplementary file 1 — Supplementary Information [file 41398_2019_448_MOESM1_ESM.docx]

**Supplementary Information**

# **Co-expression network modeling identifies key long non-coding RNA and mRNA modules in altering molecular phenotype to develop stress-induced depression in rats**

Qingzhong Wang, ^1^ Bhaskar Roy^1^, Yogesh Dwivedi^1,^*

^1^Department of Psychiatry and Behavioral Neurobiology, University of Alabama at Birmingham, Birmingham, Alabama, 35294, USA

**Keywords:** lncRNA, depression, co-expression analysis, hippocampus, stress

*Corresponding Author

Yogesh Dwivedi, Ph.D.

Elesabeth Ridgely Shook Professor

Director of Translational Research

UAB Mood Disorder Program

Department of Psychiatry and Behavioral Neurobiology

University of Alabama at Birmingham

SC711 Sparks Center

1720 7^th^ Avenue South

Birmingham, Alabama, USA

Phone: 01-205-975-8459

Fax: 01-205-975-8463

Email: [ydwivedi@uab.edu](mailto:ydwivedi@uab.edu)

**Supplementary Methods**

### *Animals*

Male Sprague-Dawley rats (Holtzman strain; age, 6-8 weeks) were purchased from Envigo Laboratories (Indianapolis, IN, USA). Rats were housed in the condition of 21 ± 1°C temperature and 55 ± 5% humidity. All rats were provided ad-libitum food and water and were acclimatized for one week prior to experiment. All behavioral experiments were performed between 8 and 10 am. The protocol to induce learned helpless behavior was approved by the Institutional Animal Care and Use Committee (IACUC) of the University of Alabama at Birmingham. All the experiments were done in 6 TC, 7 NLH, and 7 LH rats.

***Induction of learned helpless behavior***

The detailed protocol for the induction of learned helpless behavior has been described in our earlier publications^1, 2^ and is depicted in **Figure 1A**. Briefly, rats were randomly selected and were given 100 inescapable tail shocks (IS) at the intensity of 1.0 mAmp for 5 seconds. The average interval between two shocks was 60 seconds. Escape latency was tested (ET) after 24 hours. These rats were given an additional IS on day 7 and tested for escape latency on day 8 and again on day 14. Another group of rats were tested for escape latency without giving any shock and served as control (TC). Because TC rats were handled similarly as NLH and LH rats therefore, the inclusion of TC rats in the experiment helped to rule out the nonspecific effects of stress caused by restraint, tail shock, or testing. The escape latency was tested using two different trials: fixed ratio 1 (FR1) and 2 (FR2). In FR1 (5 trials) pretrial, rats were given foot shock at the intensity of 0.6 mAmp at variable time intervals. The rats had to escape the foot shock by moving from one chamber to another without coming back. In FR2 (25 trials), the rats had to cross from one chamber to the other and had to come back to the original chamber to terminate the shock. The shocks were terminated automatically after 30 sec. Escape latencies were recorded through computer generated program (Med Associates Inc., VT, USA). Based on escape latency in FR2 trial, rats were divided into two groups: learned helpless (LH, showing escape latency ≥20 seconds) and non-learned helpless (NLH, showing escape latency <20 seconds). Generally, the rats who showed LH behavior in the FR2 trial (day 2) remained LH throughout the experimental duration (day 14). We found almost equal distribution of rats among LH and non-LH groups. Twenty-four hours after the final escape latency (ET) test, rats were decapitated, and brains were dissected out. Hippocampi were isolated and flash frozen in liquid nitrogen. Tissues were stored at -80°C until they were analyzed.

***Microarray based expression profiling of lncRNAs and mRNAs***

Total RNA was isolated from rat hippocampus using Trizol method^®^ as described earlier.^3^ RNA quantity and integrity were checked based on gel electrophoresis and Nanodrop quantification. Only those RNA samples were used that had high purity (260/280) and ribosomal RNA integrity (28S:18S=2:1). Transcriptome-wide lncRNA expression was measured using one color high throughput microarray-based microarray protocol following Agilent Array platform (ArrayStar, Inc., Rockville, MD, USA). The Agilent microarray chip contains 13,611 lncRNAs and 24,626 mRNAs. Briefly, total RNA from each sample was linearly amplified and labeled with Cy3-dCTP. The labeled cRNAs were purified using the RNeasy mini kit (Qiagen, MD, USA). The concentration and specific activity of the labeled cRNAs (pmol Cy3/μg cRNA) were measured using a NanoDrop ND-1000 spectrophotometer. A total of 1 μg of each labeled cRNA was fragmented by the addition of 11 μL 10× blocking agent and 2.2 μL of 25× fragmentation buffer. The mixture was heated at 60°C for 30 minutes and 55 μL 2 × GE hybridization buffer was added to dilute the labeled cRNA. Hybridization solution (100 μL) was dispensed into the gasket slide and assembled to the Rat lncRNA expression microarray slide (4X44K). The slides were incubated for 17 hours at 65°C in an Agilent hybridization oven. The hybridized arrays were washed, fixed, and scanned using the Agilent DNA Microarray Scanner G2505C.^4, 5^

***lncRNA and mRNA data processing***

All rat lncRNA related information was collected from UCSC Known genes, Ensembl, and Refseq databases. In case of uncharacterized lncRNAs, the information related to mouse orthologue sequence was collected. Agilent Feature Extraction software (version 11.0.1.1) was used to analyze acquired array images. Quantile normalization were performed using the GeneSpring GX v12.1 software (Agilent Technologies Inc, CA, USA). After normalization by quantile methods for both mRNA and lncRNA datasets, the total number of 14605 mRNAs and 8977 lncRNAs across each group were sorted for subsequent analyses. Differentially expressed lncRNAs and mRNAs were identified through fold change using Agilent GeneSpring GX software (version 11.5.1). Fold change was calculated with log2 method. Statistical significance was determined after correcting with multiple comparisons. The false discovery rate (FDR) by Benjamini and Hochberg procedure was applied to correct for multiple testing and reported as q values.^6^ We selected the most variable transcripts with the absolute values of fold change >1.3 and p-value <0.05 as differentially expressed genes for constructing the co-expression networks.

**References**

1. Dwivedi Y, Mondal AC, Payappagoudar GV, Rizavi HS. Differential regulation of serotonin (5HT)2A receptor mRNA and protein levels after single and repeated stress in rat brain: role in learned helplessness behavior. *Neuropharmacology* 2005; **48**(2)**:** 204-214.

2. Dwivedi Y, Mondal AC, Shukla PK, Rizavi HS, Lyons J. Altered protein kinase a in brain of learned helpless rats: effects of acute and repeated stress. *Biological Psychiatry* 2004; **56**(1)**:** 30-40.

3. Roy B, Dunbar M, Shelton RC, Dwivedi Y. Identification of MicroRNA-124-3p as a Putative Epigenetic Signature of Major Depressive Disorder. *Neuropsychopharmacology* 2017; **42**(4)**:** 864-875.

4. Gopalakrishnan K, Kumarasamy S, Mell B, Joe B. Genome-wide identification of long noncoding RNAs in rat models of cardiovascular and renal disease. *Hypertension* 2015; **65**(1)**:** 200-210.

5. Roy B, Wang Q, Dwivedi Y. Long non-coding RNA-associated transcriptomic changes in resiliency or susceptibility to depression and response to antidepressant treatment. *Int J Neuropsychopharmacol* 2018.

6. Benjamini Y, Hochberg Y. Controlling the False Discovery Rate: A Practical and Powerful Approach to Multiple Testing. *Journal of the Royal Statistical Society Series B (Methodological)* 1995; **57**(1)**:** 289-300.

**Supplementary Figure Legends**

**Supplementary Figure 1:** **mRNA expression profile based WGCNA analysis in NLH vs TC groups.**  **1A.** Hierarchical cluster tree showing 4 modules of co-expressed genes including brown, turquoise, blue and yellow modules. The dendrogram was produced by average linkage hierarchical clustering of genes based on topological overlap. Color blocks denote module assignments determined by Dynamic Hybrid algorithm. **1B.** Eigengene correlation between four modules and resilience trait. Figure contains the corresponding correlation and p values (in parentheses), which represent the correlation the association between eigengens with resilience trait. Among the 4 modules, brown and turquoise modules showed positive association with the phenotypic difference while the blue and yellow modules showed negative association with the resilience (Brown module: r=0.79, p=0.001; Turquoise module: r=0.83, p=4E-04; Blue module: r=-0.8, p=9E-04; Yellow module: r=-0.66, p= 0.01).

**Supplementary Figure 2:** **Module detection and trait association in LH vs NLH group comparison.**  **2A.** A total of 417 differentially expressed transcripts were applied to perform the WGCNA analysis which yielded 3 modules. The 3 identified modules are blue, brown and turquoise and each module was assigned with a unique color under the dendrograms. **2B.** Association analysis show that all 3 modules are negatively correlated with the phenotype changes in LH vs NLH group comparison (Blue module: r=-0.79, p=8E-04; Brown module: r=-0.70, p=0.008; Turquoise module: r=-0.76, p=0.001).

**Supplementary Figure 3:** **The network analysis based on mRNA expression profile in NLH vs TC group.** **3A**. Gene significance (y-axis) versus intramodular connectivity (x-axis) is demonstrated separately in the scatterplot. In the NLH vs TC mRNA comparison, the turquoise module shows a significant correlation between connectivity and gene significance (r=0.2, p=0.0011). The other 3 modules did not reach statistical significance. **3B.** Based on intramodular connectivity, the constructed network identified key hub gene *Myl3* from the turquoise module with strong connectivity to resilient (NLH) phenotype. *Myl3* gene demonstrated both higher gene significance (Gs>0.9) and higher intramodular connectivity (Gi >0.9).

**Supplementary Figure 4:** **The network analysis based on the mRNA profile in the LH vs NLH group.** **4A.** Blue module and brown module show significant correlation between connectivity (Gi) and gene significance (Gs) (Blue module: p=0.0028; Brown module: p= 0.031). **4B**. The blue module related network demonstrates key hub mRNAs *Inexa* and *olr8*. **4C.** The network associated with brown module show key hub mRNAs *Fadd* and *Sgpi1*.

**Supplementary Figure 5:** **LH vs NLH group-based module detections and association analysis using lncRNA expression profile**. **5A**. The 3 modules containing LNCbrown, LNCblue and LNCturquoise were identified from the LH vs NLH comparison. **5B**. The figure represents the association analysis between modules and phenotype alterations in LH vs NLH groups. Brown module is positively associated with phenotype trait changes (r=0.7; p= 0.005) while the blue and turquoise module show negative correlation (Blue module: r=-0.71; p=0.004; turquoise module: r=-0.82; p=0.003).

**Supplementary Figure 6:** **Module detection and association analysis based on lncRNA expression profile in NLH vs TC group.** **6A.** In the NLH vs TC group, 5 individual modules (NTCgreen, NTCturquoise, NTCyellow, NTCred, NTCblue, NTCbrown) were identified based on lncRNA expression profile. **6B**. The NTCturquoise was identified as the most significantly associated module with resilience (r=0.8; p=0.001) followed by NTCyellow (r=0.69; p=0.009).

**Supplementary Figure 7:** **Gene significance and intramodular connectivity analysis based on lncRNA expression profile in NLH vs TC groups**. **7A.** NTCturquoise module showed strong correlation between connectivity and gene significance (p= p=0.0039). **7B.** A total of 6 lncRNAs (*XR_008967*, *XR_008566*, *XR_009175*, *XR_007909*, *XR_006115* and *XR_005783*) were identified as hub lncRNAs in the NTCturquoise module.

**Supplementary Figure 8:** **Gene significance and intramodular connectivity analysis based on lncRNA expression profile in LH vs NLH groups.** **8A.** LNCturquoise module show strong correlation between connectivity and gene significance (p=0.00017). **8B.** The 4 lncRNAs (*AY539919*, *AY562215*, *MRAK039538*, and *MRAK048306*) were identified as the hub lncRNAs in the LNCturquoise module.

**Supplementary Figure 9:** **qPCR-based expression changes of select hub genes associated with rat model of depression.** qPCR based expression validation of 4 hub genes (Rnf29, Tas2r116, Inexa and Olr8) were analyzed across three experimental groups (TC, NLH and LH) using primers mentioned in the Methods section. Gapdh normalized expression level of each transcript is presented as relative fold change. All data are the mean ± SEM. The level of significance was determined using independent-sample ‘t’ test. ‘*’ denotes significant difference between the two comparing groups. **A.** Comparison of Rnf29 (p=0.04) and Tas2r116 (p=0.02) between TC and LH groups. **B.** Comparison of Inexa and Olr8 between LH and NLH groups (data not significant).

**Supplementary Figure 10:** **Illustration of the workflow analysis followed for data generation.** An overview of the experimental plan and data analysis scheme followed in the current study. RNA samples from three groups of behaviorally tested rats (Tested control or TC, non-learned helpless or NLH and learned helpless or LH) were collected from hippocampus. After linear amplification and dye labelling of purified RNA, the entire transcriptome was profiled using Affymetrix Genechip microarrays for both lncRNA and mRNA. From the microarray data, differential expression (DEG) pattern was determined for both mRNA and lncRNAs following quantile normalization method. Based on differential expression profile, module-trait correlation was deduced using Weighted Gene Co-expression Network Analysis (WGCNA) approach. Next, functional annotation was conducted to determine hub genes and prediction was made for Gene Ontology (GO) and pathway identification. Finally, gene co-expression network was constructed for lncRNA and coding transcripts to determine their differential association with vulnerability or resiliency to stress.
