# Supplementary material for: Co-expression network modeling identifies key long non-coding RNA and mRNA modules in altering molecular phenotype to develop stress-induced depression in rats
Source: Transl Psychiatry. 2019 Apr 3;9:125. doi: 10.1038/s41398-019-0448-z (PMC6447569; doi:10.1038/s41398-019-0448-z)
Supplement: Supplementary file 11 — Primer sequences used for qPCR expression assay [file 41398_2019_448_MOESM11_ESM.docx]

| **Supplementary Table 10: Primer sequences used for qPCR expression assay** | |
| --- | --- |
| **Rat oligo** | **Sequence (5'-3')** |
| rno_Rnf29 RT F | TCTGAAGGGTGTGTAGGTGTG |
| rno_Rnf RT R | TCTGCCAGTTTGTAACCGCT |
| rno_Tas2r116 RT F | ACAGGCTGCAAAAATTGCGT |
| rno_Tas2r116 RT R | AGGTCTCTGCACTCTACGGT |
| rno_Inexa RT F | TCCAAAGTCTCATCCGCTGG |
| rno_Inexa RT R | CCACCGTTTCCTCCAGTGTT |
| rno_Olr8 RT F | TTCATCTATGCCCGACCCAG |
| rno_Olr8 RT R | AGCCAATTGCACCTCTCTGT |
