# Supplementary figures and images for: Co-expression network modeling identifies key long non-coding RNA and mRNA modules in altering molecular phenotype to develop stress-induced depression in rats
Source: Transl Psychiatry. 2019 Apr 3;9:125. doi: 10.1038/s41398-019-0448-z (PMC6447569; doi:10.1038/s41398-019-0448-z)

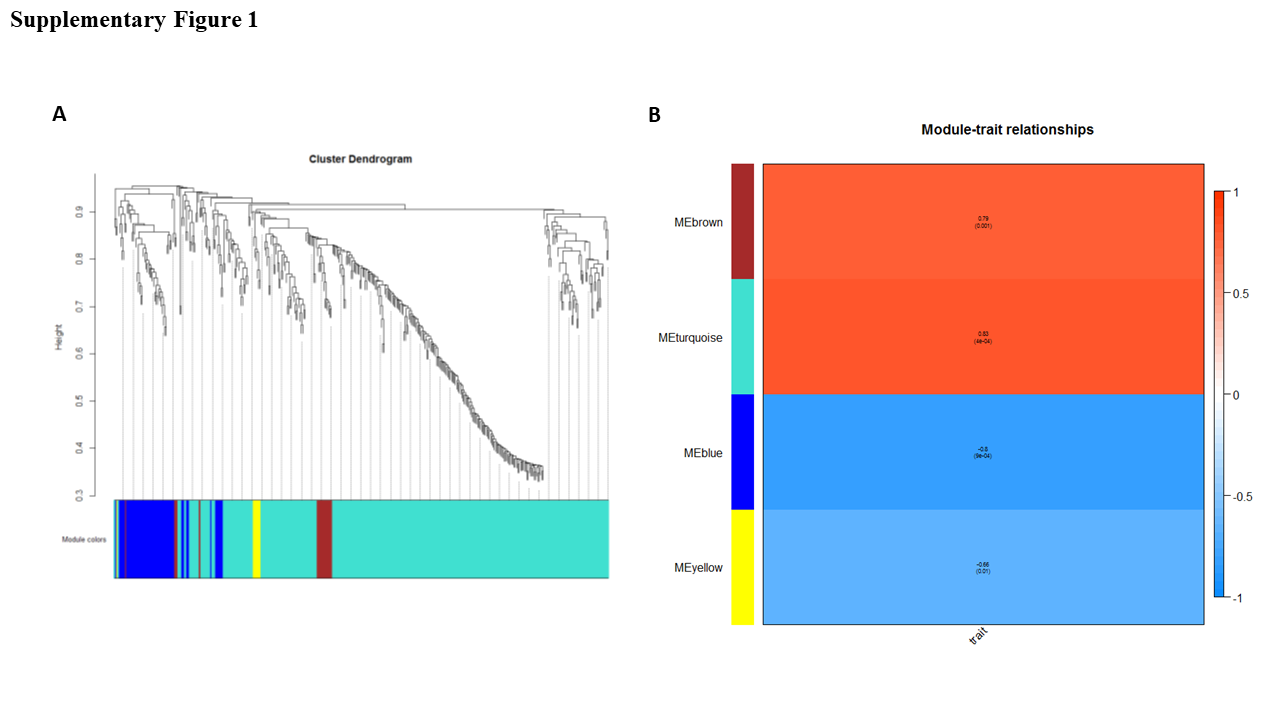

Supplement: Supplementary file 12 — Supplementary Figure 1 [file 41398_2019_448_MOESM12_ESM.tif]

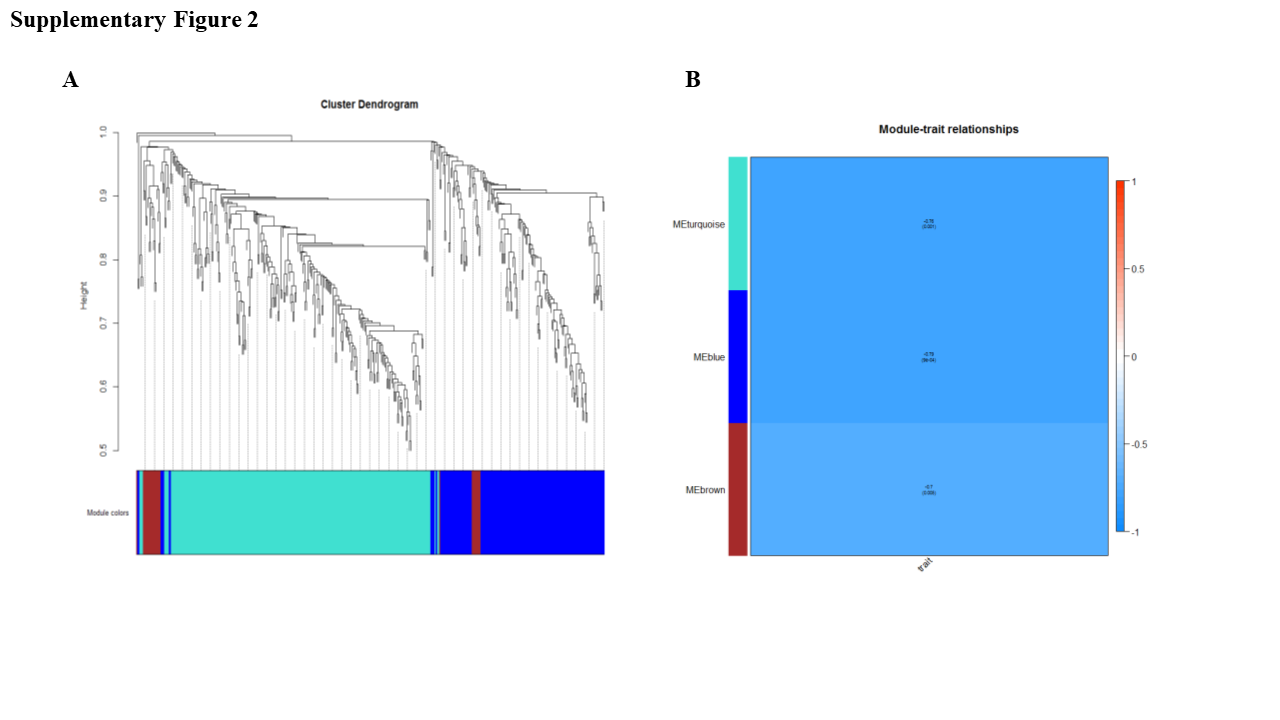

Supplement: Supplementary file 13 — Supplementary Figure 2 [file 41398_2019_448_MOESM13_ESM.tif]

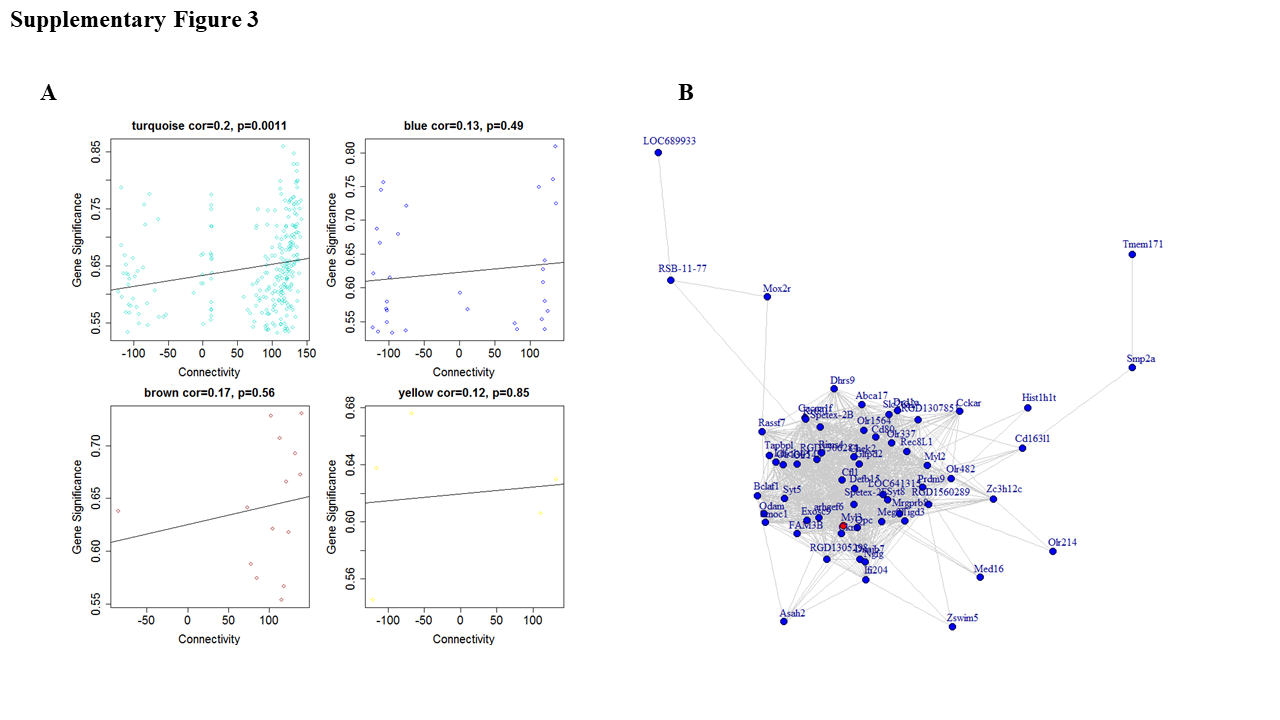

Supplement: Supplementary file 14 — Supplementary Figure 3 [file 41398_2019_448_MOESM14_ESM.tif]

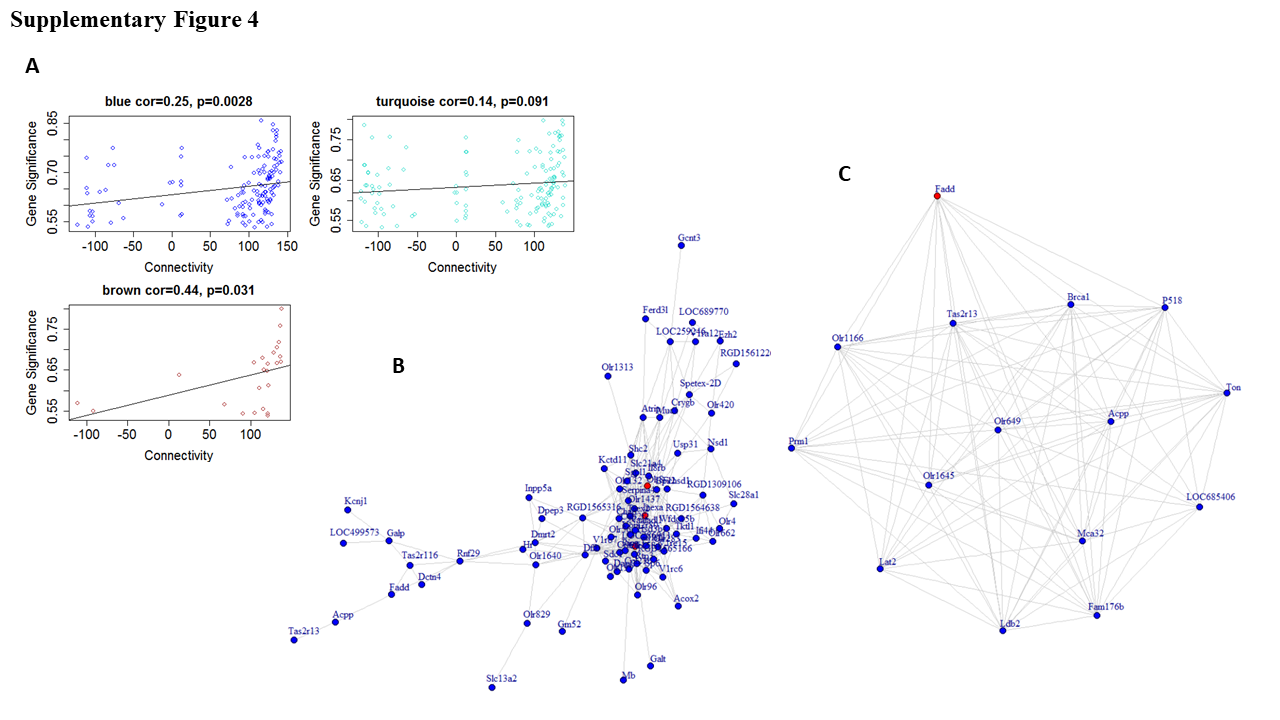

Supplement: Supplementary file 15 — Supplementary Figure 4 [file 41398_2019_448_MOESM15_ESM.tif]

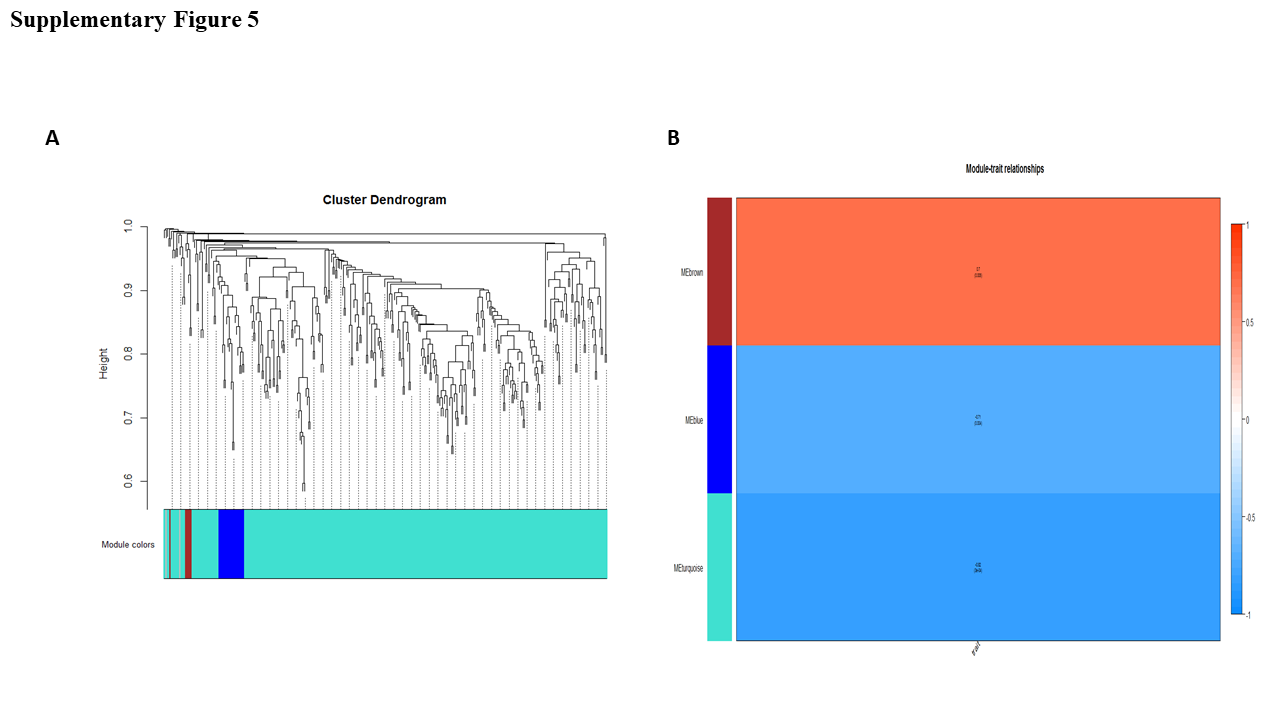

Supplement: Supplementary file 16 — Supplementary Figure 5 [file 41398_2019_448_MOESM16_ESM.tif]

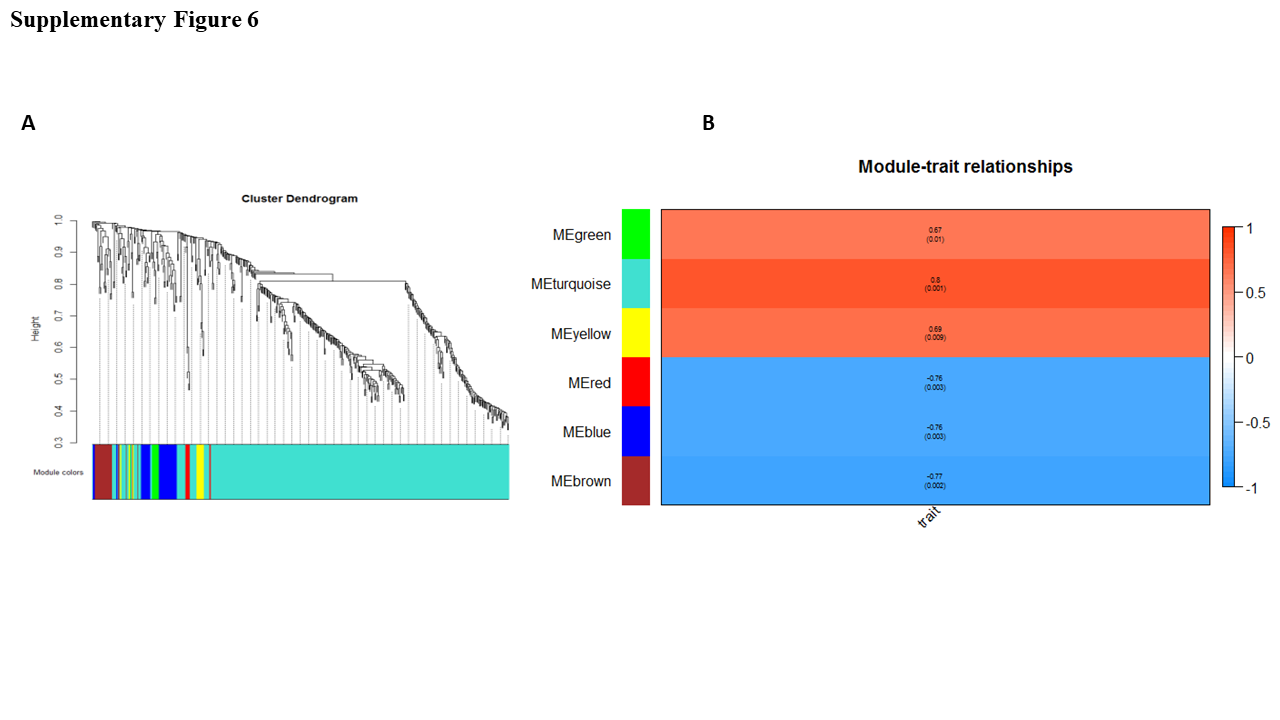

Supplement: Supplementary file 17 — Supplementary Figure 6 [file 41398_2019_448_MOESM17_ESM.tif]

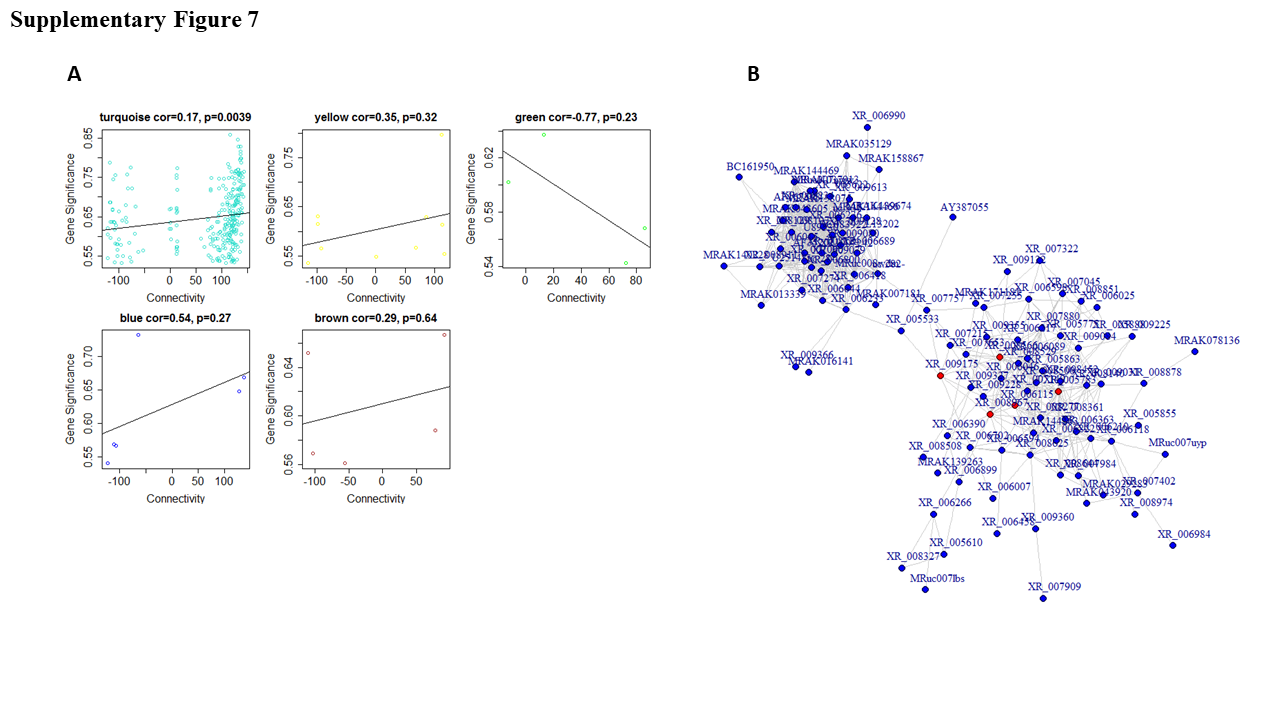

Supplement: Supplementary file 18 — Supplementary Figure 7 [file 41398_2019_448_MOESM18_ESM.tif]

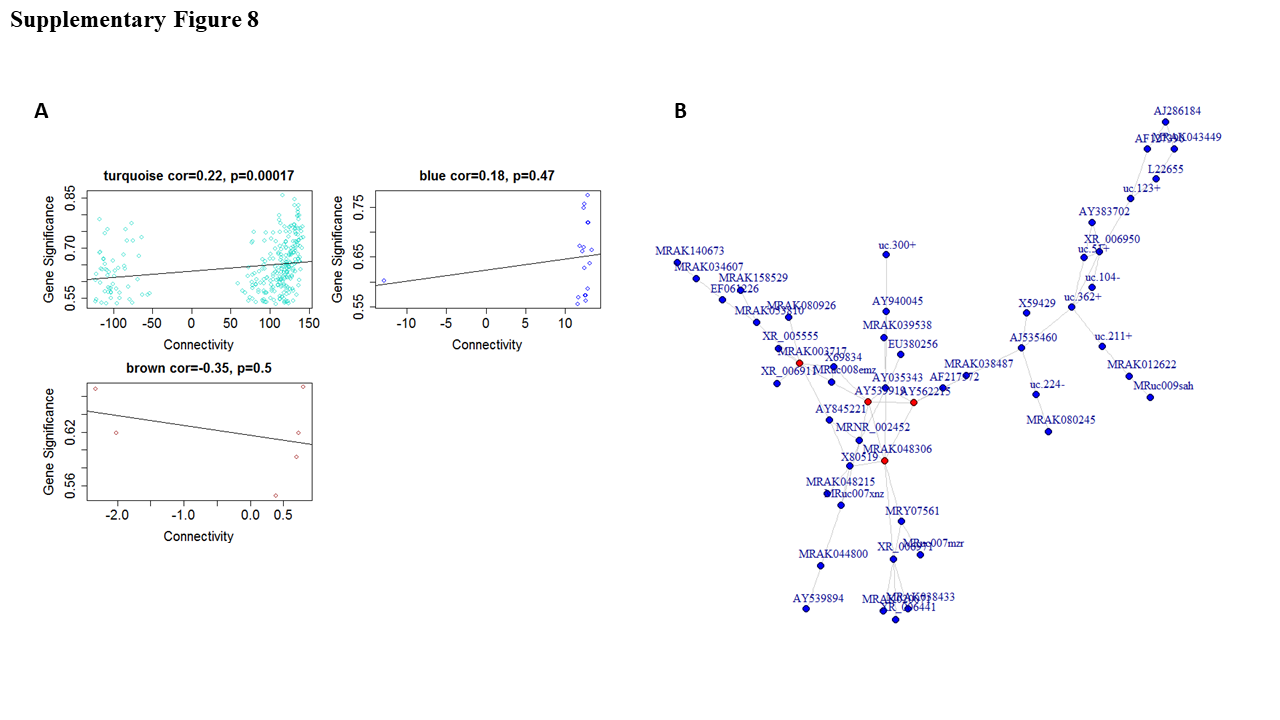

Supplement: Supplementary file 19 — Supplementary Figure 8 [file 41398_2019_448_MOESM19_ESM.tif]

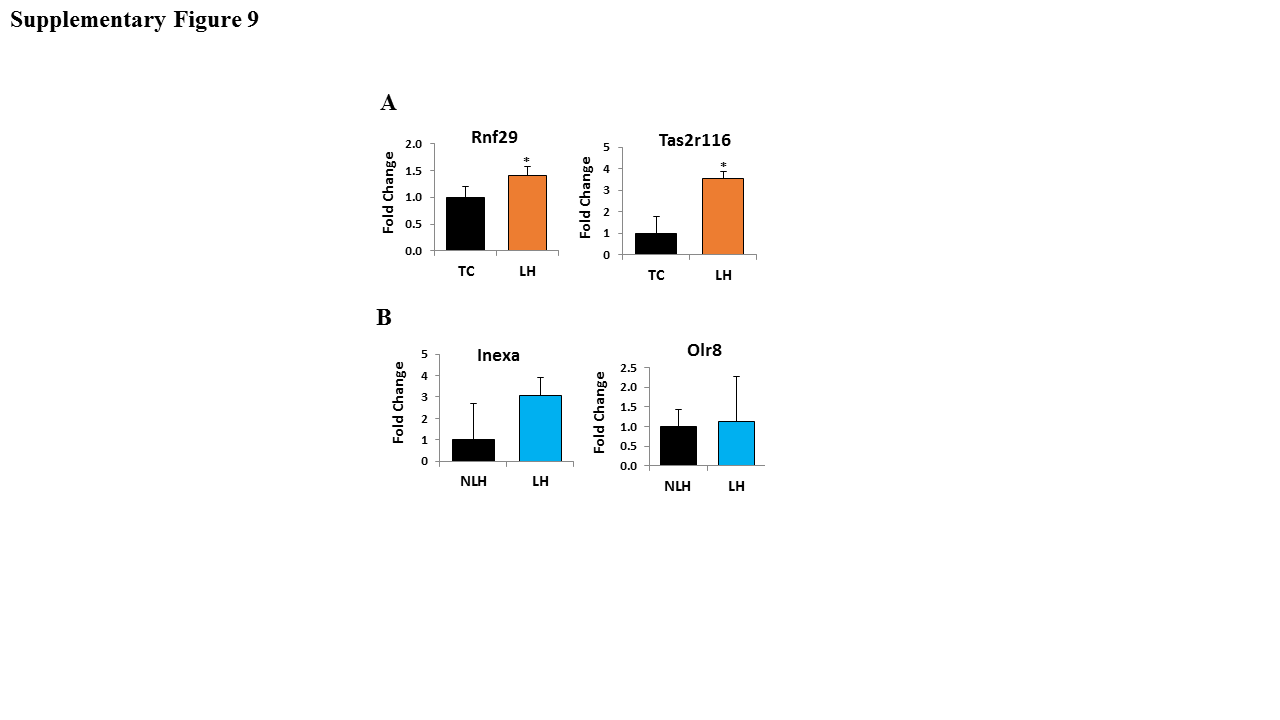

Supplement: Supplementary file 20 — qPCR-based expression changes of select hub genes associated with rat model of depression [file 41398_2019_448_MOESM20_ESM.tif]

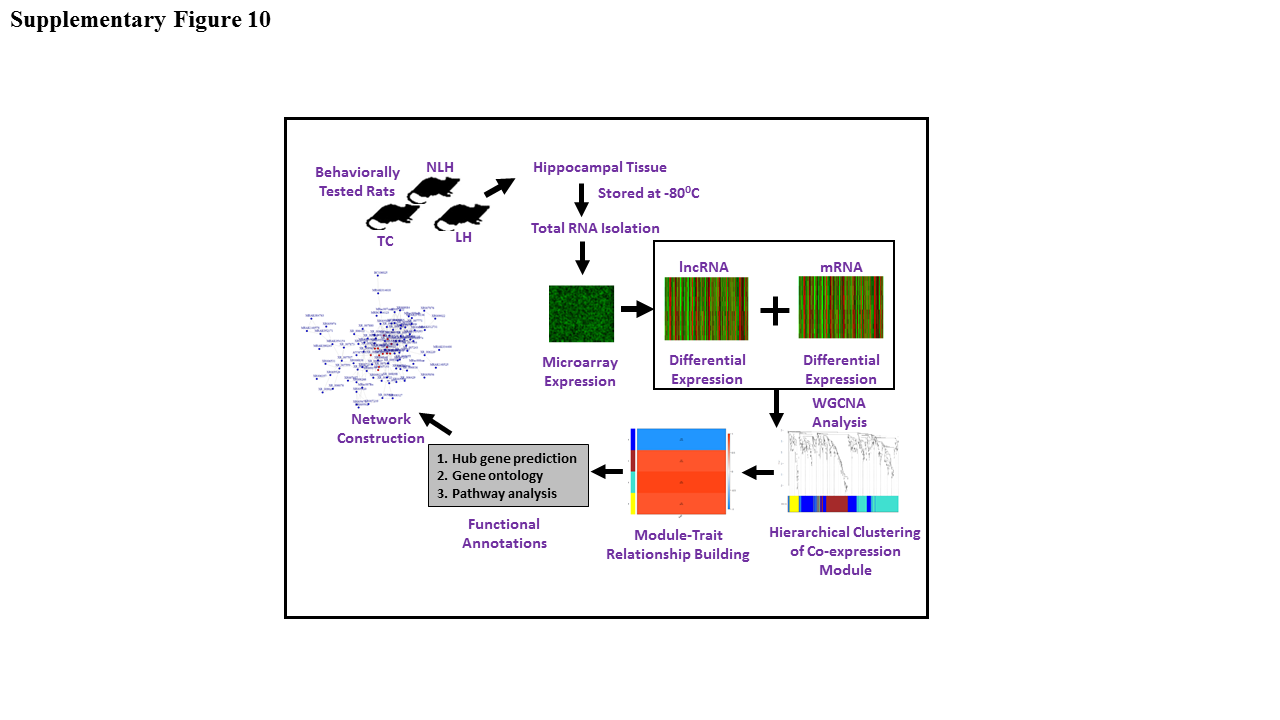

Supplement: Supplementary file 21 — Illustration of the workflow analysis followed for data generation [file 41398_2019_448_MOESM21_ESM.tif]
